# Validation of the portable Air-Smart Spirometer

**DOI:** 10.1371/journal.pone.0192789

**Published:** 2018-02-23

**Authors:** Cristina Ramos Hernández, Marta Núñez Fernández, Abel Pallares Sanmartín, Cecilia Mouronte Roibas, Luz Cerdeira Domínguez, Maria Isabel Botana Rial, Nagore Blanco Cid, Alberto Fernández Villar

**Affiliations:** 1 Department of Pneumonology, University Hospital Complex of Vigo, Pontevedra, Spain; 2 Neumo Vigo I + i. Institute of Health Research South Galicia (IISGS), Vigo, Pontevedra, Spain; 3 Department of Pneumonology, Hospital Complex of Pontevedra, Vigo, Pontevedra, Spain; Universite de Bretagne Occidentale, FRANCE

## Abstract

**Background:**

The Air-Smart Spirometer is the first portable device accepted by the European Community (EC) that performs spirometric measurements by a turbine mechanism and displays the results on a smartphone or a tablet.

**Methods:**

In this multicenter, descriptive and cross-sectional prospective study carried out in 2 hospital centers, we compare FEV1, FVC, FEV1/FVC ratio measured with the Air Smart-Spirometer device and a conventional spirometer, and analyze the ability of this new portable device to detect obstructions. Patients were included for 2 consecutive months. We calculate sensitivity, specificity, positive and negative predictive value (PPV and NPV) and likelihood ratio (LR +, LR-) as well as the Kappa Index to evaluate the concordance between the two devices for the detection of obstruction. The agreement and relation between the values of FEV1 and FVC in absolute value and the FEV1/FVC ratio measured by both devices were analyzed by calculating the intraclass correlation coefficient (ICC) and the Pearson correlation coefficient (r) respectively.

**Results:**

200 patients (100 from each center) were included with a mean age of 57 (± 14) years, 110 were men (55%). Obstruction was detected by conventional spirometry in 73 patients (40.1%). Using a FEV1/FVC ratio smaller than 0.7 to detect obstruction with the Air Smart-Spirometer, the kappa index was 0.88, sensitivity (90.4%), specificity (97.2%), PPV (95.7%), NPV (93.7%), positive likelihood ratio (32.29), and negative likelihood ratio (0.10). The ICC and r between FEV1, FVC, and FEV1 / FVC ratio measured by the Air Smart Spirometer and the conventional spirometer were all higher than 0.94.

**Conclusion:**

The Air-Smart Spirometer is a simple and very precise instrument for detecting obstructive airway diseases. It is easy to use, which could make it especially useful non-specialized care and in other areas.

## Introduction

The characteristics of portable devices including low cost, are simplicity of use, and reliability of results. Then they open new possibilities to optimize the diagnosis and monitoring of respiratory diseases. Obstructive airway disorders are of particular interest, because despite a prevalence of 5–10% in the general population, the rate of under-diagnosis reaches 80% [1,2].

It is fundamental to carry out the screening in non-specialized areas, such as primary care, in order to combat this under-diagnosis. It is essential that spirometry be performed routinely and with an appropriate quality at this level of care, but the current evidence does not support this reality[3,4].

Some of the most frequent problems include the low accessibility to spirometers in non-specialized areas, the maintenance of such devices, and the handling and interpretation of results by non-expert personnel. This has led to the design and commercialization of several portable devices that allow for the rapid collection of spirometric parameters, making them especially useful in the screening of respiratory diseases in non-specialized care areas [5,6]. However, safe use of these devices requires that they must be validated by comparing them with routine functional tests performed by trained personnel with calibrated spirometers under rigorous quality controls.

**Fig 1:**
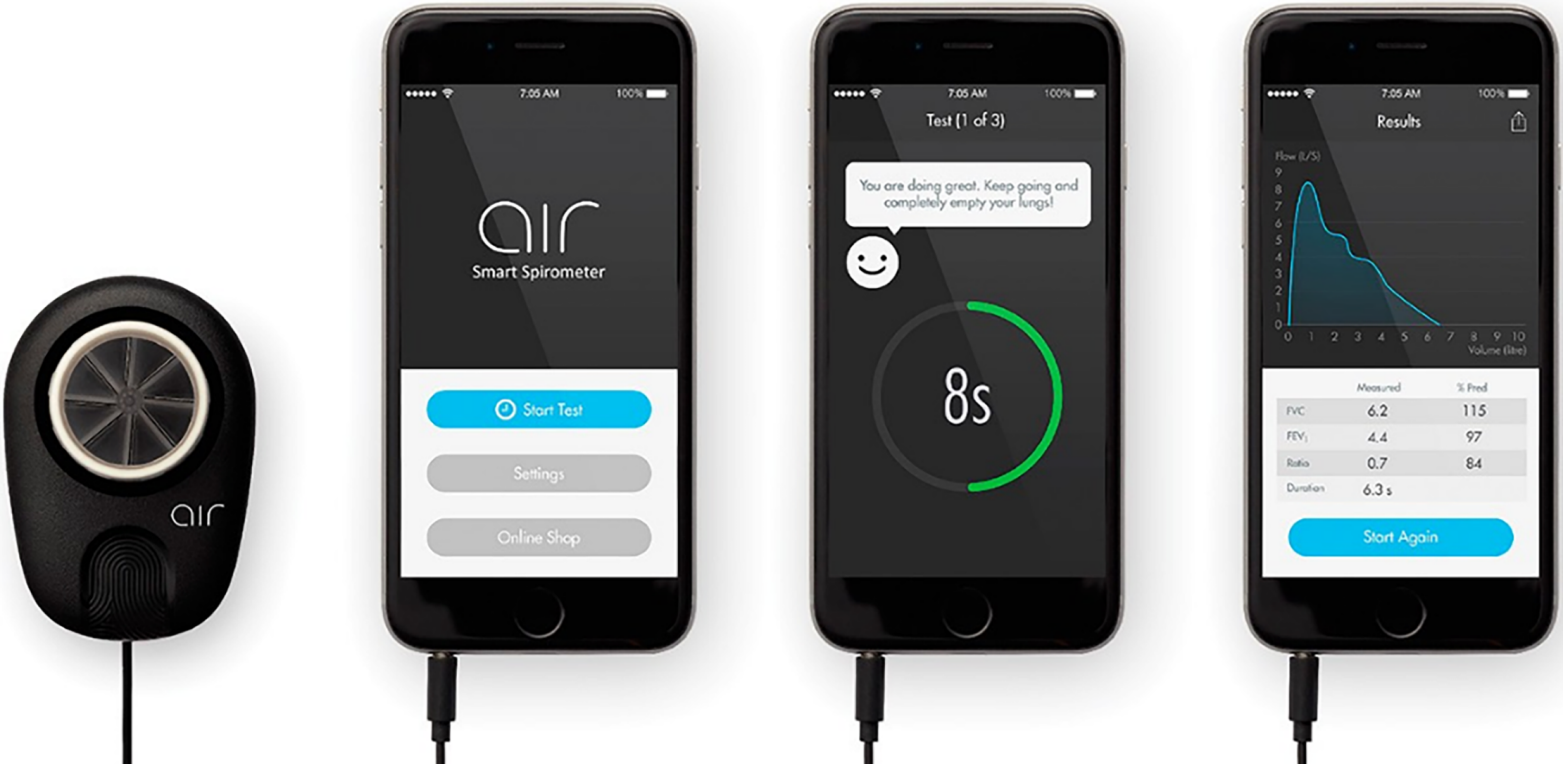
Air Smart Spirometer. After 6 seconds of exhalation the chronometer turns green.

One of the most recent of such devices marketed in our country is the Air Smart Spirometer (Pond Healthcare Innovation, Sweden). It is the first portable device accepted by the EC that allows users to visualize the results on a smartphone (Fig 1), and according to the manufacturer it is easy to use and accurate enough to reliably determine FEV1, FVC, and their ratio. To date, no work has been published examining its validity and safety as a diagnosis tool for respiratory diseases in clinical practice. Here we evaluate the concordance and relationship between the parameters obtained by the Air Smart-Spirometer and a conventional spirometer.

## Materials and methods

### Design

The study has being approved by the ethics committee on research of Vigo with the registration Code: 2017/116. This is a prospective, descriptive, and cross-sectional study performed in two centers, Hospital Álvaro Cunqueiro (University Hospital Complex of Vigo) and Hospital Complex of Pontevedra. We included 200 patients, selected consecutively from June to August 2016 from the patients assigned in the pulmonary function laboratories of both centers.

### Subjects

Each participant was informed about the study and provided written informed consent, and the study was prospectively approved by the ethical committee for clinical research in Galicia.

Patients who had any of the contraindications to make spirometry listed in the Spanish Society of Pulmonology and Thoracic Surgery (SEPAR) guidelines were excluded: haemodynamic instability, pulmonary embolism (until adequately anticoagulated), recent pneumothorax, acute haemoptysis, active respiratory infections, recent myocardial infarction or unstable angina, aneurism of the thoracic artery that has grown or is large in size (>6 cm), intracranial hypertension or acute retinal detachment [7]^.^. We also excluded patients who did not provide informed consent and those who required more than 8 maneuvers in order to be able to meet reproducibility criteria.

We consider a spirometry as acceptable if the start were rapid, without hesitation, the course of the expiratory manoeuvre were continuous, without any artefacts or evidence of coughing in the first second, the end of the manoeuvre didn´t show early or abrupt interruption. The difference between the best two acceptable VC, IC, FVC and FEV1 should be less than 0.15 [8].

### Test

All patients underwent a conventional spirometry and another with the Air Smart Spirometer device, following SEPAR requirements, to obtain acceptable and reproducible criteria[7].

Spirometry with the Air-Smart-Spirometer device was performed before or after the conventional laboratory spirometer (order was randomly chosen), in order to avoid possible biases. Measurements with both devices were carried out by trained personnel and performed in a standardized way.

### Devices

Conventional spirometry was performed on Masterlab Pneumatic-Type Spirometers with a Lilly pneumotachograph (Jaeger AG, Wurzburg, Germany). This pneumotachograph measures flow in terms of the proportional pressure drop across a resistance consisting of a very fine mesh screen and is calibrated daily with a 3 liter syringe and subject to temperature, humidity and altitude adjustment, as well as a weekly calibration of flow linearity with 1 liter syringe.

The University Hospital Complex of Vigo used Quajer Gli reference values for conventional spirometers [8].The Hospital Complex of Pontevedra used the reference values recommended by SEPAR [7].

The Air Smart Spirometer (Fig 1) is a small portable device that connects to the smartphone at the headphone jack. Its dimensions are 79 x 56 x 20 mm and it weighs 50g. It includes a lithium battery, designed to have a half-life of 2 years (or 1000 spirometries). It has a turbine mechanism (Flow Mir) to perform measurements inside the disposable single use nozzles. The approximate price of the device is 69 euros and each mouthpiece costs less than 1 euro. The device does not require calibration, but parameters of age, sex and height must be entered before spirometry is performed.

To perform spirometry, the user exhales air into the turbine. This air turns a motor, and the device registers the speed of the rotor, adapts it, and transfers the data to the smartphone application. We use two Iphone 5, to made the measurement with IOS 10.0.1 software version.

When the patient initiates exhalation, a chronometer is switched on and changes its color from red to green after 6 seconds of exhalation (Fig 1).

The portable device uses the reference values validated in the NHANES III study to calculate the percentages of FEV1 and FVC [9].

The device allows users to perform three maneuvers and it chooses the one that has obtained the best FEV1 and FVC, but it also allows users to perform individual maneuvers and visualize each curve independently. It has a flow meter which detects errors of acceptability and indicates them on the screen next to the definitive results.

### Statistical analysis

The qualitative variables were expressed by their absolute value and their percentage, and the quantitative variables are expressed as means and standard deviations (represented as mean (standard deviation). We used the mean of the difference and its 95% CI to express the differences between the parameters studied. The comparison of the quantitative variables was carried out by applying the Student’s t test for paired samples, and a p-value equal or less than 0.05 was considered statistically significant. The kappa index was used to evaluate the concordance between the two devices for the detection of obstruction (FEV1/FVC ratio <70%) as a qualitative variable. The agreement and relation between the values of FEV1 and FVC in absolute value and the FEV1/ FVC ratio measured by both devices were analyzed by calculating the intraclass correlation coefficient (ICC) and the Pearson correlation coefficient (r) respectively, and were plotted using Bland and Altman graphics and correlation plots.

The validity and safety of the Air Smart Spirometer in the detection of obstruction were determined using the usual equations, and the sensitivity, specificity, positive predictive value (PPV), negative predictive value (NPV) and positive/negative likelihood ratios (LR +, LR-) were calculated.

The sample size was based on the experience of the group in validation of other devices, estimating that the Air Smart Spirometer could present a sensitivity of 90% and a specificity of 80% in the detection of obstruction, a prevalence of the same of 40%, and an alpha error of 5% [5,6]. To obtain an accuracy of 8%, the required sample would be 162 subjects. This study followed the STARD recommendations for the evaluation of diagnostic tests [10].

## Results

A total of 200 patients (100 patients from each participating center) were consecutively enrolled in the study from the pulmonary function laboratories of the Hospital Álvaro Cunqueiro (Vigo) and the Pontevedra Hospital Complex. Of these, 110 (55%) were males. The mean age was 57 (± 14) years.

Of these patients, 64 had a previous diagnosis of COPD (32%), 53 were diagnosed with asthma (26%), 25 with Intersticial lung disease (ILD) (12.5%), 35 with Obstructive Sleep Apnea (OSA) (17.5%), 13 with bronchiectasis (6.5%), and 10 (5.5%) had dyspnea on study without previous diagnosis. Obstruction was detected by conventional spirometry in 83 (41.5%) of the samples, and mean FEV1 was 2307 ml (79.4%).

The absolute and percentage values of the different parameters measured with the conventional spirometer and the Air Smart Spirometer are shown in Table 1. The mean of the differences and the 95% CI are also included. The values obtained with the Air Smart Spirometer were significantly lower for the absolute value of FEV1 (mean difference of 8.45 ml), the absolute value of FVC (mean difference of 77 ml), and for the value of FVC percentage predicted value. The value of FEV1 compared to its reference and the FEV1/FVC ratio were significantly higher.

**Table 1:** Mean values and differences of parameters determined by conventional spirometer and Air Smart Spirometer.

	C.Spirometer^a^^,^^b^	Air SS^a^^,^^c^	*p*	Differences: Spirometer–ASS^d^
FEV1	2307 (903) ml	2298 (849) ml	<0.001	8.45ml (CI 95%: -18 to 35)
FEV1%	79 (21) %	80 (22) %	<0.001	-1.35% (CI 95%: -2.79 to 008)
FVC	3341(977) ml	3263 (955) ml	<0.001	77.95 ml (CI 95%: 38 to 117)
FVC%	90 (17) %	88 (17) %	<0.001	1.67% (CI 95%: -0.17 to 3.53)
FEV1/FVC	68 (14) %	70 (12)%	<0.001	-2.08 (CI 95%: -2.73 to -1.42)

a. Mean (standard deviation)

b. Air SS: Air Smart Spirometer

c. C. Spirometer: Conventional spirometer

d. Mean difference and 95% confidence interval of the mean.Reference Values:

Hospital Complex of Pontevedra SEPAR [7].

Hospital Álvaro Cunqueiro (Vigo) Quanjer Gli [8].

Reference Values of Air Smart Spirometer: NHANESS III [9].

The concordance and correlation between the parameters of the two devices were FEV1 (conventional spirometry) vs. FEV1 (Air Smart Spirometer) ICC = 0.98 (p <0.001), r = 0.97 (p <0.001), FVC (conventional spirometry) vs. FVC (Air Smart Spirometer) ICC = 0.98 (p <0.001), r = 0.96 (p <0.001), FEV1/FVC (conventional spirometry) vs. FEV1/FVC (Air Smart Spirometer) ICC = 0.96 (p <0.001), r = 0.94 (p <0.001) (Table 2).

**Table 2:** Concordance and correlation between the parameters measured by the Air Smart Spirometer and the conventional spirometer.

	ICC	R
FEV1	0.98 (p<0.001)	r = 0.97 (p<0.001)
FVC	0.98 (p<0.001)	r = 0.96 (p<0.001)
FEV1/FVC	0.98 (p<0.001)	r = 0.94 (p<0.001)

**Fig 2:**
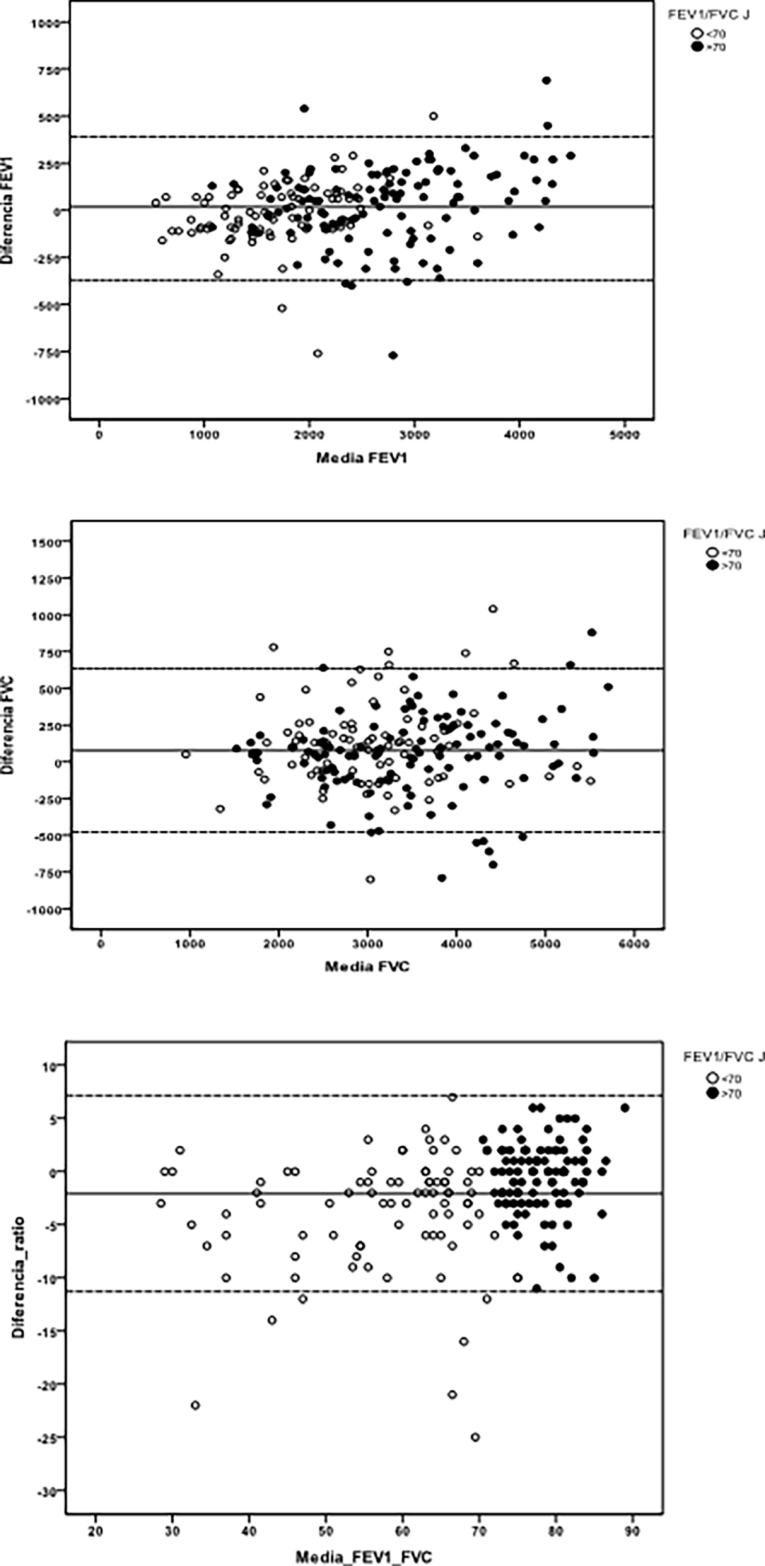
Bland and Altman graphs.

**Fig 3:**
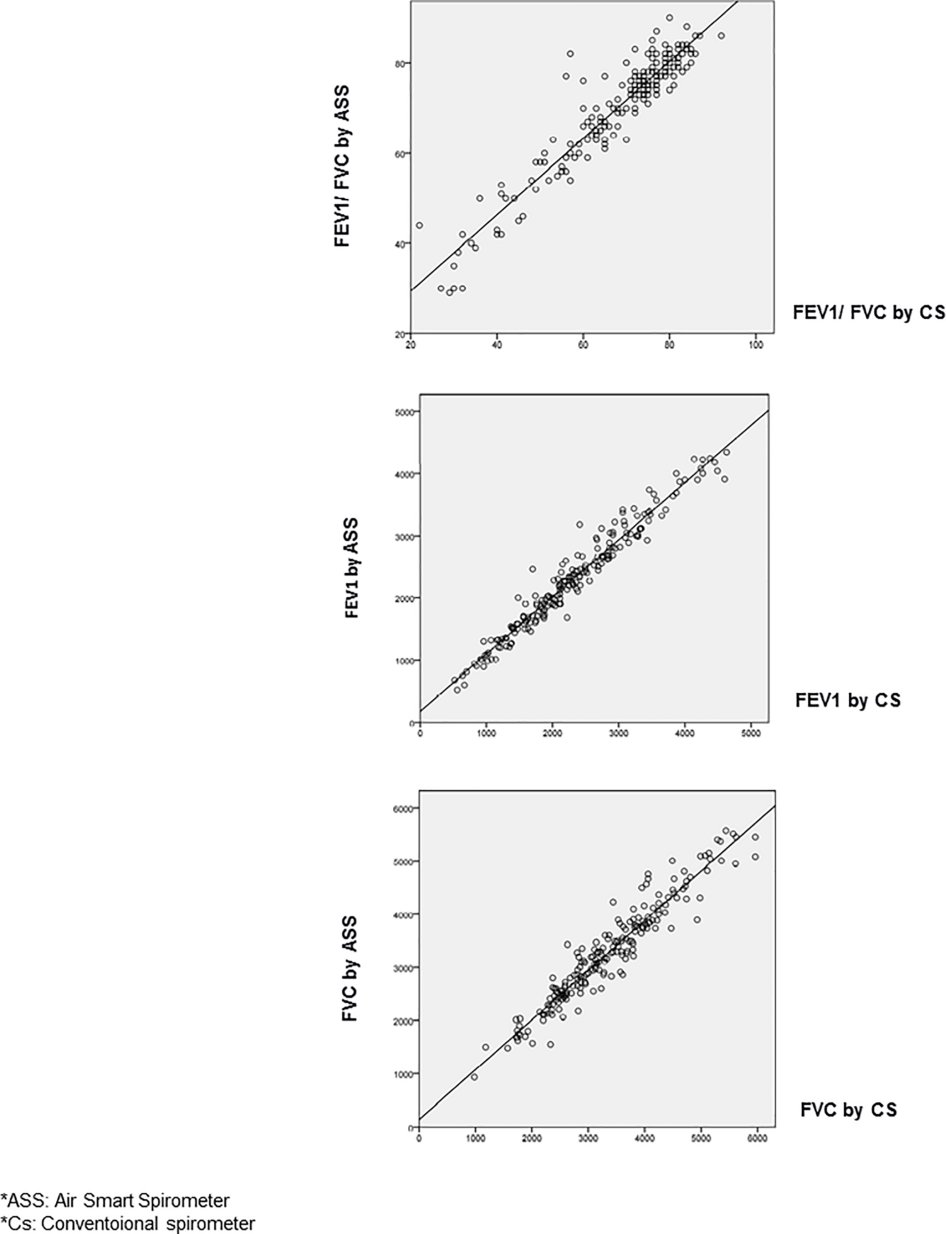
Correlation graphs.

Fig 2 presents Bland and Altman graphs, in which a homogeneous distribution can be observed independently of the FEV1 and FVC values. The correlation graphs are shown in Fig 3, which shows that this is excellent for all parameters.

A contingency table (Table 3) was created for patients diagnosed with airway obstruction with conventional Spirometry and Air Smart Spirometer, defined in both cases as FEV1/FVC ratio lower than 0.7.

**Table 3:** Contingency table of the number of subjects diagnosed with obstruction by conventional spirometer and using the Air Smart Spirometer device.

	AirSmart-Spirometer obstruction^a^		Total
	NO	YES	
Conventional spirometry obstruction ^a^			
NO	114	3	117
YES	8	75	83
**Total**	122	78	200

^(a)^ Defined by FEV1/FVC < .07

Of the 83 patients with an obstructive ratio determined with conventional spirometer, 8 (9.63%) would not have been detected with the Air Smart-Spirometer. In 2 of these the diagnosis of airflow obstruction would be ruled out if we had used the lower limit of normality instead of FEV1/FVC ratio <0.7. In 3 of these 8 patients, FVC obtained wasn´t reproducible between the portable and the conventional device, while FEV1 was. In the other 2 cases there was reproducibility between FEV1 and FVC between the two devices but not in the ratio, at the expense of a slight increase in FEV1 (the difference was <150 ml with the conventional spirometer) and a lower FVC value in the Air Smart Spirometer (the difference was <150 ml with the conventional spirometer). Regarding the degree of airflow obstruction, two of the eight false negatives had moderate obstruction and the rest had mild obstruction.

Only one patient had no correlation between the two devices in any of the analyzed values (FEV1, FVC and FEV1/FVC ratio) and was previously diagnosed with COPD.

The value of the Kappa index was 0.88 (very high). For the detection of airflow obstruction, the portable device had a sensitivity of (90.4%), specificity of (97.2%), PPV of (95.7%) and NPV of (93.7%). The (LR +) resulted in 32.29 and the (LR-) was 0.10.

## Discussion

Spirometry is an essential technique for the early diagnosis, assessment of severity, and follow-up of chronic respiratory diseases, especially those with airflow obstruction, and should be considered as a basic exploration of pulmonary function. However, although spirometry is a diagnostic technique defined as simple, noninvasive, reliable, and safe, numerous studies show the existence of problems of underuse and variability in the quality of spirometry performed [11,12]. These factors likely contribute to the underdiagnosis of chronic respiratory diseases [1,2]. In the same line of work it is necessary to implement the use of quality spirometries. To this end, training and qualification programs should be developed for the professionals responsible for carrying out spirometry. Quality and easy-to-use spirometers should be also provided [3,13].

There have been several portable spirometers validated to date, such as the Piko 6, a small device used to detect obstruction using the expiratory flow in 6 seconds (FEV6) to replace FVC in obstruction detection [14–16]. The Vitalograph COPD-6 device was validated and proved to be a useful tool for screening for obstructive respiratory diseases from a non-specialized area using a FEV1/FEV6 ratio < 0.8 [5,6]. Most of the validated portable devices do not allow users to show the flow/volume or volume/time loops, making it difficult to assess possible bias in the technique. Others such as the EasyOne Spirometer make a measurement of flows through ultrasounds. This allows for the display of the volume/flow loop, but this device is quite expensive and the results of a test on volunteers showed correlation in FVC but not in FEV1[17].

Given current technological advances the increase in COPD and aging of the population provided for the next years, rapid and accurate diagnostics are needed. Computing and medicine is a current example of the process of the integration of scientific disciplines and constitutes a nexus between science, technology, and society. Over the last decade, health-related technologies associated with mobile devices (mHealth) have evolved dramatically in an attempt to address specific needs and improve access to diagnostic testing.

According to the manufacturer, the Air Smart Spirometer offers great accessibility, since its dimensions allow for easy handling, it does not require calibration, and the nozzles are disposable and of individual use. It can be connected to any Smartphone device after downloading an application called "Air". Unlike other portable devices, this allows the visualization of the spirometric loop, giving a greater value to the results and making it possible to critically evaluate the maneuvers performed. It could even facilitate the diagnosis of pathologies of the upper airway through the morphology of the loop [7]. The device itself detects errors in acceptance criteria that it reports in the display of the results. In our study, early termination errors (less than 6 seconds) were often detected when performing the maneuver with the Air Smart Spirometer, but this was a device problem (that stopped counting the time despite continuing the expiratory flow) already addressed by the Pond Healthcare Innovation through a software update.

Our study is the first to analyze the validity and safety of the Air Smart Spirometer device, and we conclude that it presents excellent validity for the diagnosis of obstruction when compared with the gold standard (conventional spirometry). The concordance and relation of the FEV1 and FVC parameters were optimal. We used the absolute values (not the percentages) to make this comparison, because the theoretical values used in the two participating centers were different. We did not find any differences when we analyzed the concordance of the results from each center separately. The greatest difference in the measurement was in the FVC, which was 77.95 ml (95% CI: 38 to 117). This could be related to the spirometric technique or to pneumotachograph used, as this is the parameter known to show more discordance when comparing studies performed by different professionals [4]. In spite of this, the limits included in the confidence interval fulfill reproducibility criteria. The high concordance of these measurements makes it a useful tool for the early detection of chronic respiratory diseases, and may find greater value in the field of non-specialized care because normal values rule out airway obstruction with acceptable safety.

Thanks to its dimensions, it could be used to carry out rapid appraisals in consultation. The Air application allows users to save the results and connect directly to health-related applications, such as the Health application on Apple devices, providing the ability to monitor patients at home.

There are several limitations to the present study. First, the study was carried out by Respiratory Nurse Specialist, a fact that may limit external validity in other areas where professionals have less experience. Further studies are needed in other areas (such as primary care, pharmacies, and emergencies) to assess their validity at this level [6]. On the other hand, the cutoff point used to detect airflow obstruction was FEV1/FVC ratio <0.7, this index is controversial because of its possible inaccuracy, especially at the age limits (both young and old). Currently most authors support the idea of using the lower limit of normality as reference to avoid under-diagnosis in young subjects and over-diagnosis in older adults [18]. However, here we considered FEV1/FVC ratio <0,7 more appropriate to simplify the diagnosis to validate the measurements of a new device.

In conclusion, the Air Smart Spirometer device is a simple, manageable, and very precise device that could be used for the screening and diagnosis of chronic respiratory diseases and also for individual monitoring at the patient's home.
